# Typical Aroma of Merlot Dry Red Wine from Eastern Foothill of Helan Mountain in Ningxia, China

**DOI:** 10.3390/molecules28155682

**Published:** 2023-07-27

**Authors:** Lijun Sun, Zhong Zhang, Hongchuan Xia, Qingchen Zhang, Junxiang Zhang

**Affiliations:** 1 School of Agriculture, Ningxia University, Yinchuan 750021, China; sunlijun2022@126.com (L.S.); hongchuanxia@126.com (H.X.); 2 School of Life Sciences, Ningxia University, Yinchuan 750021, China; zhangzhongnxu@126.com; 3 Yinchuan Wine Industry Development Service Center, Yinchuan 750021, China; 4 College of Pharmacy, University of Florida, Gainesville, FL 32610, USA; qingchen.zhang@ufl.edu; 5 School of Food and Wine, Ningxia University, Yinchuan 750021, China; 6 China Wine Industry Technology Institute, Yinchuan 750021, China

**Keywords:** aroma characteristics, volatile organic compounds, Merlot, grape wine, geranyl isovalerate

## Abstract

Aroma is an important aspect of wine quality and consumer appreciation. The volatile organic compounds (VOCs) and olfactory profiles of Merlot dry red wines from the Eastern Foothill of Helan Mountain (EFHM) were analyzed using gas chromatography-mass spectrometry and quantitative descriptive analysis. The results showed that Merlot wines from EFHM were characterized by intense flavors of drupe and tropical fruits compared with the Gansu region. Nineteen VOCs were defined as essential compounds contributing to the aroma characteristics of the Merlot wines through gas chromatography–olfactometry/mass spectrometry and odor activity value analysis. Predominantly, geranyl isovalerate, which contributed to the herbal odors of the Merlot wines, was detected in the grape wine of EFHM for the first time. The addition experiment revealed that geranyl isovalerate influenced the aroma quality of wine by increasing herbal odors and enhancing the olfactory intensities of tropical fruits. These results are helpful for further understanding the aroma of Merlot wines from EFHM and improving the quality of wine aromas.

## 1. Introduction 

Aroma is an essential sensory aspect of wine and is highly valued by connoisseurs and consumers. Volatile organic compounds (VOCs) are the chemical basis of the aroma profiles of wine, which exhibit a nonrandom distribution pattern across different regions and contribute to the distinctiveness of wine [1,2,3]. The VOCs deriving from grape berries, alcoholic fermentation, and the aging process can be defined as primary, secondary, and tertiary aromas of wine, respectively [4]. Although hundreds of VOCs have been identified in grapes and wines, only a few help mold the typicality of wine flavor [5,6,7].

Some VOCs are found in low concentrations in wine, but they may have a considerable effect on the aroma quality of wine because of their low sensory threshold at the level of ng·L^−1^ [4,8]. Nevertheless, it is reported that only a subset of volatiles interacts with the olfactory receptors in the human nose to cause an aroma perception in the brain. A sensory-guided analysis, such as gas chromatography olfactometry (GC-O), revealed that some aroma compounds can be separated from the majority of odorless volatiles [9]. VOCs with odor activity values (OAV) > 1 or odor intensity values greater than 3 in GC-O are generally considered to contribute to the overall aroma of a wine and are considered the key aroma substances in wine [10,11]. Mayr et al. [12] analyzed Syrah wines from Australia using GC-O, quantitation, and aroma reconstitution techniques. According to OAV, ethyl octanoate, ethyl hexanoate, ethyl-3-methylbutanoate, ethyl-2-methylbutanoate, ethyl acetate, β-damascenone, 3-methyl butanoic acid, eugenol, and cis-whisky lactone were found to be the most significant contributors to Syrah wine aroma. Herderich et al. [13] found that rotundone, an oxygenated sesquiterpene, was the key aroma compound responsible for the ‘pepper’ aroma in Australian Syrah wines. Zhao et al. [14] characterized the aroma of Syrah wines from China. According to GC-O, ethyl-2-methylpropanoate, ethyl-3-methylbutanoate, 3-methylbutyl acetate, 2,3-methyl-1-butanol, ethyl hexanoate, ethyl octanoate, 2-phenethyl acetate, methional, 3-methylbutanoic acid, hexanoic acid, octanoic acid, β-damascenone, guaiacol, 2-phenylethanol, trans-whiskylactone, 4-ethylguaiacol, eugenol, 4-ethylphenol, and sotolon were detected as the key aroma compounds. Ling et al. [15] characterized the aroma of Dornfelder wines from three production regions of China through aroma reconstitution, omission tests, and descriptive analysis. They claimed that terpenoids could be regarded as key aroma compounds directly contributing to the floral odors of Chinese Dornfelder wines. Lyu et al. [16] reconstructed the aroma outline of Marselan wine from the Xinjiang region through 21 key aroma compounds with OAV > 1. Lan et al. [17] utilized omission experiments to reveal that 3-mercaptohexanol, (E)-β-damascenone, furaneol, ethyl cinnamate, γ-octalactone, γ-decalactone, and γ-hexalactone have a significant influence on the Petit Manseng wine typicality. 

The Eastern Foothill of Helan Mountain (EFHM) in Ningxia is one of the most representative viticultural zones of China [18]. In 2003, EFHM wines were awarded the “National Geographical Indication Products of Wine” by the Geographical Indications Committee of China [19]. Merlot (*Vitis vinifera* L.) is a preferred red grape variety that originated in Bordeaux in France and has been cultivated worldwide [20]. Although Merlot is one of the main grape varieties in EFHM, there are few reports about the aroma characteristics and essential VOCs of Merlot dry red wines in EFHM. In this study, we collected 72 bottles of unaged Merlot wines from EFHM (Ningxia), as well as two other well-established Chinese wine regions, Xinjiang (XJ) and Gansu (GS). By comparing their VOC contents and olfactory characteristics, we aimed to reveal the aroma typicality of Ningxia Merlot wines.

## 2. Results and Analysis

### 2.1. Aroma Characteristics of Merlot Wines

**Figure 1:**
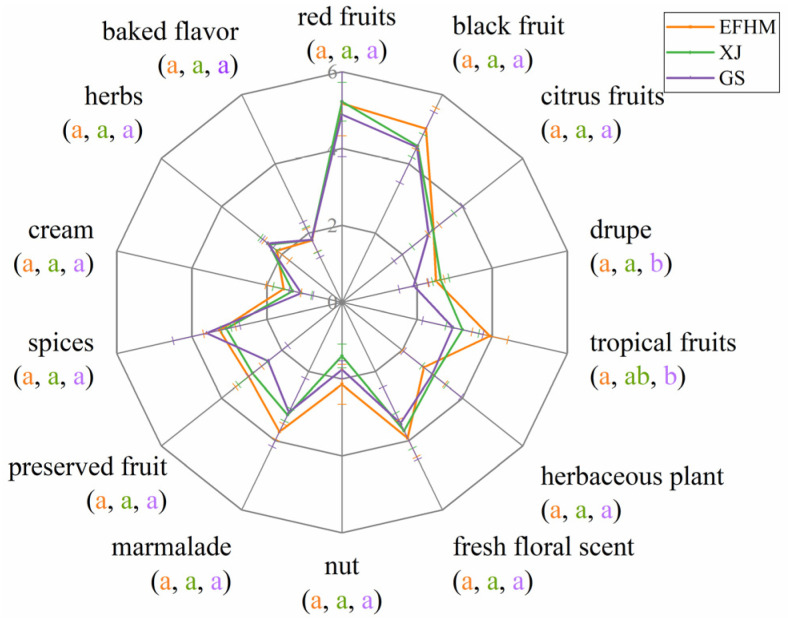
Aroma profiles of Merlot wines from different regions. EFHM, Eastern Foothill of Helan Mountain; XJ, Xinjiang; GS, Gansu. Different letters for each aroma attribute indicate significant differences (Duncan’s test, *p* < 0.05), with the color and order of the letters corresponding to the legend.

The aroma profiles of Merlot dry red wines from different regions were calculated based on the intensity values obtained from the quantitative description analysis (Figure 1). The typical flavor of Merlot wines was described as red and black fruits. The intensity scores of drupe odor in EFHM and XJ wines were significantly higher than in GS wines. The aroma intensity of tropical fruits in EFHM wines was much greater than GS. However, there was no significant difference in other aroma characteristics among the three regions.

### 2.2. VOCs in Merlot Wines

**Figure 2:**
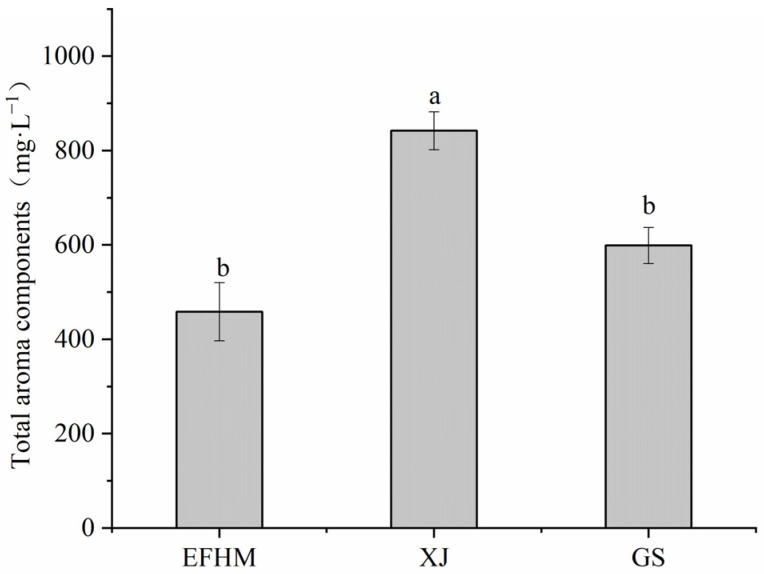
Total VOC contents of Merlot wines from different regions. EFHM, Eastern Foothill of Helan Mountain; XJ, Xinjiang; GS, Gansu. Different letters on the bars indicate significant differences (Duncan’s test, *p* < 0.05).

The variation in aroma characteristics arises from different concentrations of VOCs [8]. The headspace solid-phase micro-extraction coupled with gas chromatography-mass spectrometry (HS-SPME-GC-MS) technique detected 51 VOCs in the 72 Merlot wine samples from different regions (Table 1). Each region was considered a distinct entity, and the content of VOCs was aggregated accordingly, as depicted in Figure 2. The total VOC amount was significantly lower in EFHM and GS wines compared with XJ. Nonetheless, the sensory characteristics of the wine are not solely reliant on the cumulative presence of VOCs, but are also influenced by the specific arrangement of distinct categories [21].

**Figure 3:**
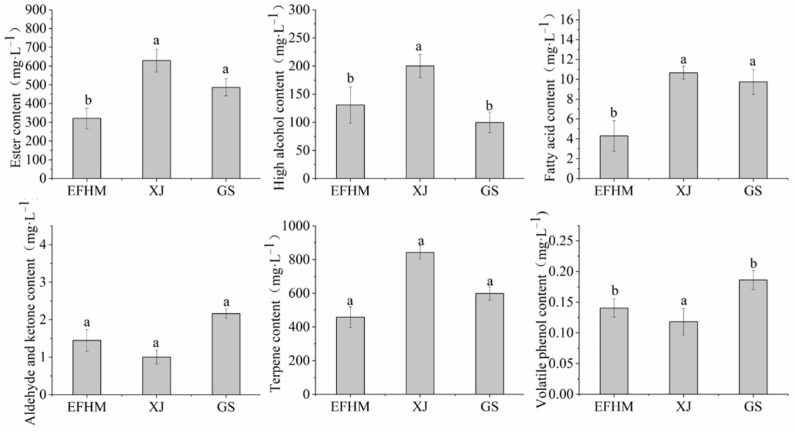
Different categories of VOCs in Merlot wines from different regions. EFHM, Eastern Foothill of Helan Mountain; XJ, Xinjiang; GS, Gansu. Different letters on the bars indicate significant differences (Duncan’s test, *p* < 0.05).

The 51 VOCs were grouped into six types: esters (29), higher alcohols (8), fatty acids (5), carbonyl compounds (7), terpenes (1), and volatile phenols (1), according to the similarity of chemical structures (Figure 3). Although most esters were below the olfactory thresholds, they may contribute to the fruity characteristics of wine due to the synergistic effects [22,23]. Unfortunately, the total content of esters in EFHM Merlot dry red wine was significantly lower than in the other two regions.

Higher alcohols in wine are produced through two pathways during alcoholic fermentation, the Harris pathway of glycolysis and the Ehrlich pathway in amino acid degradation [24]. Studies have shown that higher alcohols can increase the aroma complexity of wine when it is below 300 mg·L^−1^ [24]. From Figure 3, the alcohol contents of all the Merlot wine samples were less than this level, and the dissimilarity among regions was not significant.

Fatty acids in wine are mainly produced during the alcoholic fermentation stage, which is a by-product of fatty acid metabolism [25]. Fatty acids consist of straight-chain and branched-chain structures depending on whether branches exist in the carbon chain. Wine has cheese-like fragrances from straight-chain fatty acids, while rotten and oily odors come from branched-chain fatty acids [26]. The fatty acid content of Merlot dry red wine in EFHM was significantly lower than in the XJ and GS wines (Figure 3).

Aldehydes and ketones may contribute distinctive flavors to grape wine because their olfactory thresholds are typically 100–10,000 times lower than the corresponding alcohols [26]. Examples include methional with boiled potato smells, nonanal with citrus aromas, and diacetyl with butter odors [26,27]. From Figure 3, the dissimilarity of aldehydes and ketones among regions was not significant.

Terpenes are aroma compounds deriving from grape berries, which are synthesized through the methylerythritol 4-phosphate pathway and the mevalonate pathway [28]. Terpenes contribute floral and fruity characteristics to wine, whose concentrations in EFHM wines were compatible with other regions.

Excessive volatile phenols impair the fruity intensity of wine by bringing about smells described as “animal”, “stable flavor”, “leather”, “spicy” [29], and “medicinal” [30]. However, volatile phenols are believed to enhance the aroma complexity at a concentration below 420 μg·L^−1^ [30,31]. The total content of volatile phenols in Merlot dry red wine (Figure 3) in EFHM was significantly higher than in XJ wines.

**Table 1:** Aromatic substances of Merlot dry red wines from various regions.

Aroma Substance	Concentration (mg·L^−1^)			LRI	LRI *	Quantitative Method
	EFHM	XJ	GS			
Ethyl acetate	6.94 ± 1.29 b	9.21 ± 2.19 a	8.15 ± 1.52 ab	885	886	Q
Isoamyl acetate	6.43 ± 2.43 b	17.7 ± 4.61 a	10.52 ± 6.42 b	1127	1112	Q
Hexyl acetate	0.07 ± 0.1 b	0.93 ± 1.12 a	0.83 ± 0.6 a	1261	1266	Q
Phenyl ethyl acetate	0.57 ± 0.43 b	3.11 ± 0.82 a	1.3 ± 1.25 b	1821	1791	Q
Ethyl butyrate	0.55 ± 0.18 a	0.8 ± 0.37 a	0.72 ± 0.19 a	1032	1025	Q
Ethyl hexanoate	18.5 ± 6.51 b	31.69 ± 9.77 a	27.47 ± 10.15 ab	1236	1228	Q
Ethyl caprylate	167.78 ± 34.87 b	286.89 ± 73.34 a	278.09 ± 134.52 a	1424	1429	Q
Ethyl pelanoate	0.56 ± 0.16 b	0.97 ± 0.21 a	0.97 ± 0.22 a	1520	1524	Q
Ethyl caprate	95.37 ± 18.41 b	153.61 ± 39.98 a	185.82 ± 89.88 a	1638	1633	Q
Diethyl succinate	8.33 ± 8.89 a	8.84 ± 5.33 a	6.14 ± 4.77 a	1686	1666	Q
Ethyl-9-decenoate	1.18 ± 0.37 a	9.85 ± 11.12 a	17.82 ± 39.59 a	1708	1680	Q
Ethyl laurate	9.18 ± 9.03 b	11.26 ± 3.05 b	20.74 ± 7.83 a	1835	1834	Q
Ethyl palmitate	0.41 ± 0.27 b	1.01 ± 0.25 a	1.03 ± 0.59 a	2243	2119	Q
Ethyl lactate	0.79 ± 0.66 b	1.58 ± 0.28 a	1.15 ± 0.26 ab	1334	1335	Q
Methyl octanoate	0.66 ± 0.26 a	0.98 ± 0.25 a	0.61 ± 0.52 a	1378	1382	Q
Methyl Salicylate	0.11 ± 0.25 a	0 ± 0 a	0 ± 0 a	1775	1771	Q
n-Decyl alcohol	0.11 ± 0.25 a	0.49 ± 0.76 a	0.26 ± 0.41 a	1778	1760	Q
Isopentyl alcohol	53.99 ± 9.83 ab	65.38 ± 18.06 a	44.29 ± 14.91 b	1200	1209	Q
2,3-Butanediol	0 ± 0 b	0.13 ± 0.2 a	0 ± 0 b	1545	1568	Q
1-Hexanol	2.15 ± 0.72 b	3.57 ± 1.39 a	2.05 ± 1.04 b	1371	1351	Q
Benzyl alcohol	0.41 ± 0.23 b	0.94 ± 0.22 a	0.18 ± 0.27 b	1869	1857	Q
2-Phenylethanol	45.79 ± 38.83 b	129.53 ± 52.97 a	49.79 ± 20.96 b	1844	1889	Q
β-Damascenone	0.11 ± 0.06 ab	0.08 ± 0.12 b	0.24 ± 0.23 a	1820	1820	Q
Nonanal	0 ± 0 b	0 ± 0 b	0.41 ± 0.64 a	1388	1385	Q
Decanal	0 ± 0 b	0 ± 0 b	0.4 ± 0.33 a	1494	1481	Q
Benzaldehyde	0 ± 0 b	0 ± 0 b	0.14 ± 0.22 a	1507	1508	Q
Phenylacetaldehyde	0.01 ± 0.03 a	0 ± 0 a	0 ± 0 a	1630	1632	Q
Hexanoic acid	0.47 ± 0.31 b	1.49 ± 0.8 a	0.72 ± 0.57 b	1851	1858	Q
n-Octanoic acid	2.84 ± 0.94 b	7.17 ± 2.43 a	5.97 ± 3.12 a	2011	1900	Q
Styrene	0.64 ± 0.14 b	1.11 ± 0.2 a	0.91 ± 0.77 ab	1248	1243	Q
Ethyl myristate	0.21 ± 0.12 b	0.72 ± 0.1 a	0.91 ± 0.45 a	2057	2024	SQ
Ethyl-2-hexenoate	0.18 ± 0.09 a	0.16 ± 0.14 a	1.39 ± 2.8 a	1357	1345	SQ
Ethyl heptanoate	0.17 ± 0.05 b	0.42 ± 0.33 a	0.19 ± 0.08 b	1336	1327	SQ
Methyl hexanoate	0 ± 0 a	0 ± 0.01 a	0.01 ± 0.01 a	1178	1179	SQ
Methyl decanoate	0.44 ± 0.11 b	0.63 ± 0.11 ab	0.71 ± 0.39 a	1593	1583	SQ
Isobutyl Decanoate	0 ± 0 b	0 ± 0 b	0.1 ± 0.16 a	1749	1747	SQ
Dibutyl suberate	0.05 ± 0.07 ab	0.15 ± 0.23 a	0 ± 0b	1601	1553	SQ
Isobutyl caprylate	0.06 ± 0.09 b	0 ± 0 b	0.39 ± 0.3 a	1550	1541	SQ
Isoamyl Octanoate	1.06 ± 0.25 b	2.76 ± 0.77 a	2.35 ± 1.32 a	1655	1650	SQ
Isoamyl Hexanoate	0.83 ± 0.28 b	1.56 ± 0.28 a	1.04 ± 0.6 b	1469	1448	SQ
Butyl lactate	0.03 ± 0.08 a	0 ± 0 a	0 ± 0 a	—	960	SQ
Amyl butyrate	0.01 ± 0.01 a	0.02 ± 0.03 a	0 ± 0 a	1321	1259	SQ
2-Ethylhexyl Butyrate	0 ± 0 b	0.07 ± 0.11 a	0 ± 0 b	—	1381	SQ
2-Methyl-1-propanol	1.07 ± 0.21 b	1.46 ± 0.26 a	1.47 ± 0.57 a	1090	1089	SQ
2,6,8-Trimethyl-4-nonanol	0.28 ± 0.15 a	0.33 ± 0.31 a	0.14 ± 0.11 a	—	1553	SQ
Valeric acid	0.09 ± 0.21 a	0 ± 0 a	0 ± 0 a	1731	1445	SQ
Ethyl isoamyl succinic acid	0.49 ± 0.55 ab	1.15 ± 0.75 a	0.38 ± 0.58 b	1892	1836	SQ
Capric acid	0.40 ± 0.27 c	1.21 ± 0.34 b	2.15 ± 1.33 a	2237	2050	SQ
2,6,8-trimethyl-4-Nonanone	0.43 ± 0.6 a	0.32 ± 0.33 a	0.76 ± 1.18 a	—	1388	SQ
2-Methyl-4-undecanone	0.76 ± 0.8 ab	0 ± 0 b	1.02 ± 0.88 a	—	1401	SQ
2,4-Di-tert-butylphenol	0.14 ± 0.1 a	0.18 ± 0.02 a	0.12 ± 0.1 a	2321	2139	SQ

The data are presented in the form of “mean ± standard deviation”. In the rows, different letters represent significant differences between samples (Duncan’s test, *p* < 0.05). LRI: linear retention indices on the DB-Wax column obtained from the NIST Chemistry WebBook (https://webbook.nist.gov/ (accessed on 23 July 2023)). LRI *: linear retention indices calculated according to the retention times of C8-C20 n-alkanes and the retention time of each compound on the DB-Wax column [32]. The compound marked “Q” indicates that it was quantified using a standard curve, while the compound labeled “SQ” indicates it was semi-quantified.

### 2.3. Aroma Markers of EFHM Merlot Wines

#### 2.3.1. Perceivable VOCs in GC-O/MS Analysis

Among the VOCs with GC-O/MS signals (Table 2), ethyl isovalerate, isoamyl acetate, 3-benzyl-2-heptanone, isoamyl alcohol, ethyl-3-methylvalerate, ethyl hexanoate, and six other aroma substances have noticeable fruity aromas. Phenethyl alcohol, diethyl succinate, and other two aroma substances possess a pronounced floral fragrance. 1,3-Diacetoxy-2-propyllaurate, β-violet alcohol, and 4-hydroxyphenethyl alcohol are substances having a rich sweet scent. Ethyl caprylate, 2,3-butanediol, acetoin, octanoic acid, and hexanoic acid are fragrant components with creamy smells. Predominantly, geranyl isovalerate was first identified in EFHM Merlot wines. Geranyl isovalerate is an herb-like odorant commonly found in *Argyreia nervosa*, a plant in the orchid family. Overall, the descriptions of these olfactory markers can support the results of the aroma profile analysis in Figure 1.

#### 2.3.2. VOCs above Olfactory Threshold

A substance with OAV higher than 1 has an essential effect on the aroma quality of wine, and a higher OAV indicates more essential contributions to wine aroma. Nine VOCs, including isoamyl acetate (OAV = 204.99), ethyl hexanoate (OAV = 35.29), ethyl caprylate (OAV = 231.97), ethyl caprate (OAV = 645.41), ethyl laurate (OAV = 9.33), phenyl ethyl acetate (OAV = 7.30), phenethyl alcohol (OAV = 6.23), octanoic acid (OAV = 8.98), and β-Damascenone (OAV = 2416.22), are extremely important for the aroma of Merlot wine in EFHM (Table 3).

#### 2.3.3. Reconstruction of Aroma of Merlot Wine

**Figure 4:**
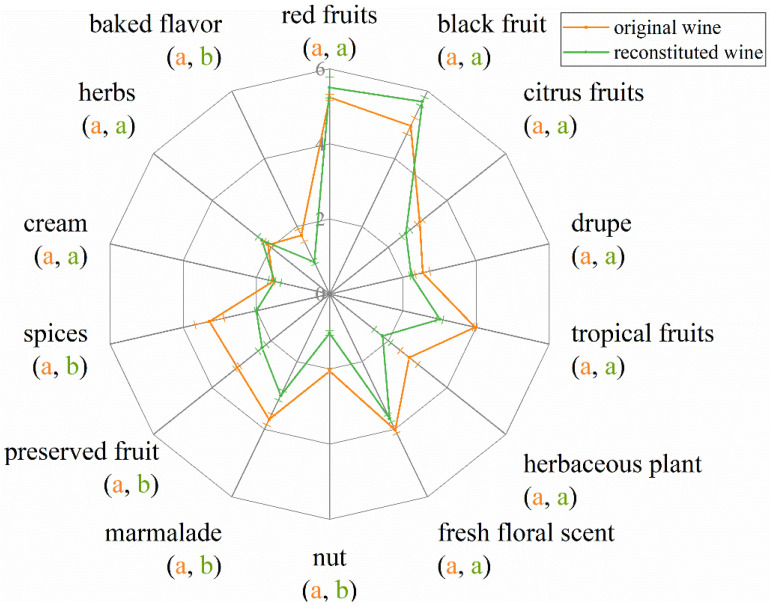
Aroma profile analysis of the reconstructed Merlot wine. Different letters for each aroma attribute indicate significant differences (Duncan’s test, *p* < 0.05), with the color and order of the letters corresponding to the legend.

Considering the sample HL2 having aroma characteristics close to the average of the EFHM wine samples, it was chosen as a control in the reconstruction experiment. The aroma of Merlot dry red wine was imitated using 19 standards of VOCs (Table 4) with OAVs larger than 1, and with olfactory intensities greater than 3. The reconstructed wine was assessed using the QDA method. The aroma profiles on fruity, flower, cream, herbs, and herbaceous plants can be reproduced by the 19 key VOCs, but the smells of nut, marmalade, preserved fruit, spices, and baked flavor of the reconstructed sample deviated from the original wine (Figure 4).

#### 2.3.4. Omission Tests for Geraniol Isovalerate

To investigate the significance of the geraniol isovalerate contribution to Merlot wine, an omission model was prepared to compare with the reconstitution model by a triangle test. Table 5 showed that the omission of geraniol isovalerate, responsible for the herbal odors of wine, was successfully perceived by all panelists with high significance (*p* ≤ 0.05).

#### 2.3.5. Addition Experiments for Geraniol Isovalerate

**Figure 5:**
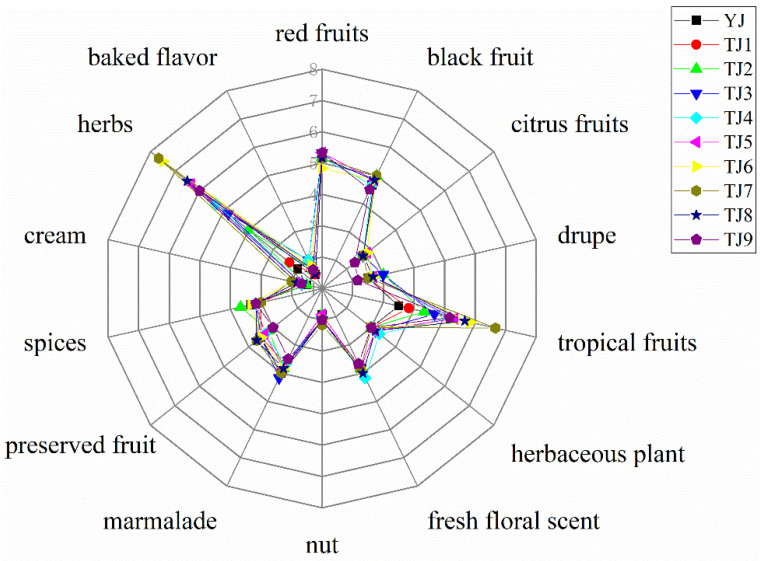
Effects of different additive amounts of geranyl isovalerate on the aroma profiles of Merlot wines. The following represents the concentrations of geraniol isovalerate added to the original wine HL2: YJ, 0 μg·L^−1^; TJ1, 10 μg·L^−1^; TJ2, 20 μg·L^−1^, TJ 3, 30 μg·L^−1^, TJ 4, 40 μg·L^−1^, TJ 5, 50 μg·L^−1^, TJ 6, 60 μg·L^−1^, TJ 7, 0.7 μg·L^−1^, TJ 8, 80 μg·L^−1^, TJ 9, 90 μg·L^−1^.

**Figure 6:**
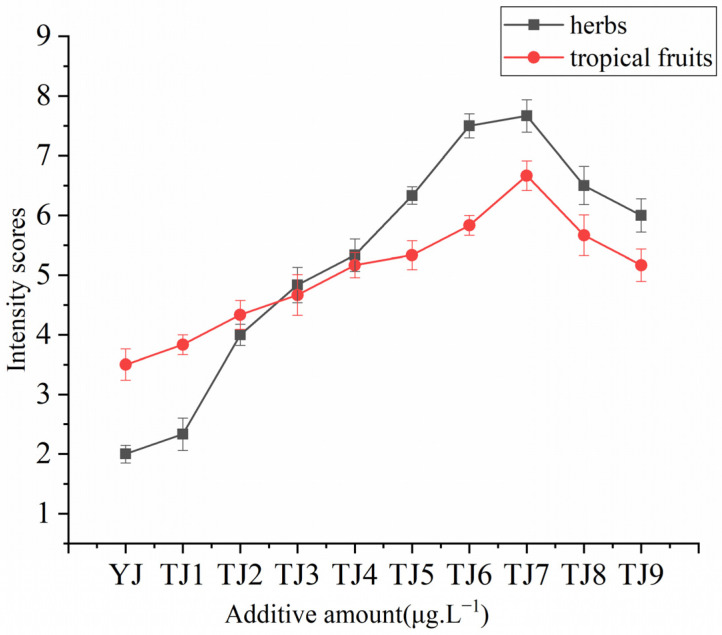
Effects of different additive amounts of geranyl isovalerate on the herbs and tropical fruit aroma intensity values of Merlot wines. The following represents the concentrations of geraniol isovalerate added to the original wine HL2: YJ, 0 μg·L^−1^; TJ1, 10 μg·L^−1^; TJ2, 20 μg·L^−1^, TJ 3, 30 μg·L^−1^, TJ 4, 40 μg·L^−1^, TJ 5, 50 μg·L^−1^, TJ 6, 60 μg·L^−1^, TJ 7, 0.7 μg·L^−1^, TJ 8, 80 μg·L^−1^, TJ 9, 90 μg·L^−1^.

The sensory threshold for geraniol isovalerate was 60 μg·L^−1^. To evaluate the perceptual interactions in Merlot wine, geraniol isovalerate at various concentrations was added according to the threshold value and the contents in real wines. Aroma addition experiments highlighted that geraniol isovalerate had significant effects on the herbs and tropical fruit aroma intensity of Merlot wine at the concentrations measured in a Merlot wine sample (Figure 5). The aromatic intensity of herbs and tropical fruits exhibited consistent changing patterns, initially increasing and reaching its peak around TJ7, followed by a subsequent decrease (Figure 6).

## 3. Materials and Methods

### 3.1. Wine Sample Collection

Seventy-two bottles of Merlot dry red wine (2020 vintage) were collected from 12 wineries located at the EFHM, XJ, and GS wine-producing regions (Table 6). None of these wines underwent oak barrel aging. After completing alcoholic fermentation and malolactic fermentation, the wines were stabilized for a period of 3 months in stainless steel storage tanks. Subsequently, they were filtered, bottled, and promptly sent to the laboratory for analysis.

### 3.2. Conventional Analysis

Alcoholicity (%, *v*/*v*), dry matter (g/L), titratable acidity (expressed as g/L of tartaric acid), residual sugar (g/L), and volatile acidity (expressed as g/L of acetic acid) were measured according to the OIV Compendium of International Methods of Wine and Must Analysis (2008). The pH was measured with a PHS-2F pH meter (INESA, Shanghai, China). From Table 7, all the wine samples were dry wines (residual sugar less than 4 g·L^−1^). The EFHM wines exhibited higher alcohol content and lower pH. The volatile acidity content of all samples was below 0.5 g/L, thus avoiding any potential interference in subsequent olfactory sensory analysis.

### 3.3. Sensory Analysis 

The sensory panel consisted of six wine professionals (three males and three females, 20–30 years old). All the panelists were trained for four weeks before the formal sniffing. At the end of the training, a set of wine samples was provided to the panel for description and discussion. A total of 14 aroma descriptors (red fruits, black fruits, citrus fruits, drupe, tropical fruits, herbaceous plant, fresh floral scent, nut, marmalade, preserved fruit, spices, cream, herbs, and baked flavor) were identified for QDA analysis [5,8,11,15]. 

The formal experiment was conducted in a blind sniffing in a standard wine tasting room (ISO 8589-1998) at 16 °C, using standard wine glasses (ISO 3591-1997) covered with aluminum foil, thereby preventing the panelists from being influenced by the appearance of the wine and affecting the evaluation of its aroma. Each panelist was required to rate the intensity of each descriptor on a scale of 0–10 twice (i.e., two repetitions: once in the morning and once in the afternoon). Each repetition consisted of six sessions, during which the panelist compared the aromas of 12 wine samples from different regions. Although all 12 samples were presented simultaneously within each session, the panelists were asked to take a break of 30–60 s between smelling every two glasses to prevent olfactory fatigue.

### 3.4. Volatile Organic Compounds Analysis 

#### 3.4.1. GC-MS Analysis 

The headspace solid-phase microextraction combined with a 7890B gas chromatography-7000D mass spectrometer (Agilent Technologies, Santa Clara, CA, USA) was used to extract and analyze the aromatic substances [37]. The divinylbenzene/carboxen/polydimethylsiloxane (DVB/CAR/PDMS) fiber (50/30 μm, 1 cm) was previously conditioned in a baking out unit according to the manufacturer’s recommendations (250 °C × 10 min). Then, 1.5 g of NaCl, 5 mL of the wine sample and 10 μL of 4-methyl-2-pentanol (1.008, 3 g/L) were added to the headspace vial. The mixture was preheated at 40 °C for 5 min and extracted using the fiber at 40 °C for 30 min on a CTC PAL autosampler (CTC Analytics, Zwingen, Switzerland). The fiber was desorbed at 240 °C for 10 min in the splitless mode. The carrier gas was high-purity helium (purity ≥ 99.999%), with a flow rate of 1 mL·min^−1^. The initial oven temperature for the DB-WAX column (film thickness of 30 m × 0.25 mm id × 0.25 μm; J&W Scientific, Folsom, CA, USA) was 40 °C, held for 5 min, then raised to 97 °C for 7 min at 3 °C·min^−1^, then raised to 120 °C at 2 °C·min^−1^, raised to 150 °C at 3 °C·min^−1^, and finally raised to 220 °C at 8 °C·min^−1^ for 10 min. The temperature of the transferred line was set at 230 °C. In the MS detector, the full scan mode (35–300 *m*/*z*) and electron ionization source were used, with a source temperature of 230 °C and electron energy of 70 eV. 

#### 3.4.2. GC-O/MS Analysis 

GC-O/MS analysis was performed using the 7890B GC-7000D MS equipped with an olfactory detection port (ODP-4, Gerstel, Mülheim, Germany). EFHM wine samples were extracted by the liquid-liquid extraction method. Twenty milliliters of wine were added to a 50-mL centrifuge tube, 15 mL of dichloromethane and 2 g of NaCl were added, and 80 μL of 4-methyl-2-pentanol (18 μg·mL^−1^) was added as a standard internal reference. The sample was vortexed for 10 min, sonicated for 20 min, and then left at 4 °C for stratifying. The organic phase was collected with a disposable needle tube, and 4 g of sodium sulfate was added for dehydration overnight. Finally, the extract was concentrated with pure nitrogen and stored at −20 °C for analysis.

In a splitless mode, one microliter of the enriched extract was delivered into the front inlet of the 7890 B gas chromatography. The oven temperature for the DB-WAX column was originally held at 40 °C for 5 min, then escalated to 230 °C at a rate of 5 °C/min, and kept at that temperature for 5 min. A flow of 1 mL·min^−1^ of helium was used as the carrier gas. The split ratio between ODP and MS was 1:1. The temperature of the sniffing transmission line and the sniffing port were 260 °C and 220 °C, respectively, and the humidifier flow rate was 12 mL·min^−1^. The six panelists were asked to record the intensity and smell of each VOC on a 1–4 scale (1, weak; 2, medium; 3, strong; 4, extremely strong).

#### 3.4.3. Qualitative and Quantitative Analysis

VOCs were identified through the NIST 17 standard spectral library and further verified with linear retention indices (LRIs) of Alkanes C8 to C20 (Sigma-Aldrich, Shanghai, China) on the DB-Wax column. Stock solutions of standards were prepared volumetrically in absolute ethanol and stored at −20 °C until dissolved in synthetic wines (15% (*v*/*v*) of alcohol, 5 g·L^−1^ of tartaric acid, and pH 3.6) to prepare the calibration data. For quantification, 10-point calibration curves were prepared for each compound by employing a progressive dilution method with a twofold decrease at each step (Table S1). The compounds without standards were semi-quantified, as described by Xia et al. [38].

### 3.5. Aroma Reconstruction Experiments

The VOCs used for aroma reconstruction were only those that simultaneously satisfy the following two conditions: (1) OAV greater than 1.0 and (2) intensity determined by GC-O no less than 3.0 [8,9,10,11,16]. They were added to a synthetic wine (prepared as describe in Section 3.4) according to their concentrations in real wines. The panelists were asked to evaluate the intensities of 14 aroma attributes of the reconstructed wine on a scale from 0 (not perceivable) to 10 (strongly perceivable).

### 3.6. Aroma Omission Experiments

An omission trial was performed to determine the role of geranyl isovalerate in the herbal odors of Merlot wines. The geranyl isovalerate omission model was compared with two completely reconstructed samples, as described in Section 3.5, using a triangle test, and the samples were assigned random numbers [16]. A group of expanded panelists consisting of ten males and ten females, aged between 20 and 30 years, were asked to sniff these samples and indicate one that exhibited differences.

### 3.7. Aroma Addition Experiments

In the addition experiment, we opted for HL2 as the base wine, primarily due to its VOC content closely resembling the average of all collected Merlot wines from the EFHM. The range of added geranyl isovalerate varied between 10 and 90 μg·L^−1^. The 4 mL mixture of geranyl isovalerate and HL2 or HL2 alone were kept in an odor-proof container (5 mL) until evaluation [39]. The expanded panelists assessed the 14 aroma attributes described in Section 3.3 on a scale of 0 (absent) to 10 (highly intense). The assessments were conducted in triplicate.

### 3.8. Measurement of Odor Threshold

A synthetic wine (prepared as described in Section 3.4) served as the base wine for the odor threshold tests. Ten concentrations of geranyl isovalerate ranging from 0 to 90 μg·L^−1^ were evaluated. Each concentration was presented as a set of three glasses and arranged in ascending order of potency. The accuracy of the expanded 20 panelists was evaluated through a triangle test. Correct responses were recorded and plotted against the concentration, and the concentration at which 50% of the judges answered correctly was designated the odor threshold [40].

### 3.9. Statistical Analysis

Data calculations were performed using Microsoft Excel 2016 software (Microsoft Office, Redmond, WA, USA). One-way ANOVA and the Duncan test were applied to determine the variances of basic oenological parameters, volatile aroma components, and sensory scores. The bar chart and radar map were drawn with Origin 2017 software (OriginLab Corporation, Northampton, MA, USA).

## 4. Conclusions

In this study, the sensory description analysis and GC-MS analysis were used to investigate the aroma of Merlot wines in three regions of China under different terroirs. The Chinese Merlot wines were described as having intense flavors of black and red fruits. The aroma components of Merlot wine showed differences among different regions. The contents of esters and fatty acids in wine samples from EFHM were the lowest. Due to wine typicality related to terroir, this result could be an indication of different specific soil, topography, climate, landscape characteristics, and biodiversity features of each winemaking region [41,42].

Based on GC-O/MS analysis and OAV analysis, nineteen odor-active compounds were selected to reconstruct the aroma profile of EFHM Merlot wines. The results of GC-O/MS analysis, omission tests, and reconstruction experiments further indicated that geranyl isovalerate contributed to the herb aroma. Geranyl isovalerate was detected for the first time in Merlot wine from EFHM. The initial documentation of geranyl isovalerate occurred in a medical journal publication in September of 2021, where its biological properties were elucidated. Upon ingestion by the human body, this compound can directly interact with the epithelial cells of the gastrointestinal tract and exhibit anti-cancer properties [43]. The additional experiments showed that perceptual interactions among the key aroma compounds in Merlot wine vary with different geranyl isovalerate concentrations. However, the factors behind the shifting patterns in the intensity of aromas found in herbs and tropical fruits remain uncertain and warrant additional investigation. Additionally, these factors may interact synergistically with other compounds [39], highlighting the need for further research in this area.

## Figures and Tables

**Table 2:** Results of olfactory analysis of Merlot wine aroma.

Compound Name	Aroma Description	Aroma Intensity						Average Value	Qualitative Method	LRI	LRI *
		EFHM 1	EFHM 2	EFHM 3	EFHM 4	EFHM 5	EFHM 6				
N-propanol	Alcohol	3.7	2.0	3.7	2.3	3.7	2.3	2.9	LRI, MS, O	1038	1041
Ethyl isovalerate	Fruity	2.3	3.7	2.0	2.3	2.7	4.0	2.8	LRI, MS, O	1072	1058
1,3-Diacetoxy-2-propyllaurate	Sweet	0.0	1.3	0.0	0.0	0.0	0.0	0.2	LRI, MS, O	——	1702
3,7,11-Trimethyl-1-dodecanol	Caramel	0.0	0.0	0.0	0.0	1.3	0.0	0.2	LRI, MS, O	——	1576
4-Octadecylal	Chocolate	0.0	0.0	0.0	0.0	0.0	0.0	0.0	LRI, MS, O	1700	1702
Isobutanol	Fusel oil	0.0	0.0	0.0	0.0	0.0	0.0	0.0	LRI, MS, O	1090	1089
Isoamyl acetate	Fruity, banana	3.0	2.0	2.7	3.3	3.3	3.7	3.0	LRI, MS, O	1127	1112
N-Butanol	Fusel oil	0.0	0.0	0.0	1.3	0.0	0.0	0.2	LRI, MS, O	1147	1147
3,4-Dimethyl-2-hexanol		0.0	0.0	0.0	0.7	0.0	0.0	0.1	LRI, MS, O	——	1890
3-Benzyl-2-heptanone	Fruity	3.7	1.3	2.0	2.3	2.2	2.1	2.3	LRI, MS, O	——	1437
Isoamyl alcohol	Fruity, bitter almonds	0.0	2.7	0.0	0.0	0.0	0.0	0.4	LRI, MS, O	1200	1209
Ethyl -3-methylvalerate	Fruity	0.0	0.3	0.0	0.0	0.0	0.0	0.0	LRI, MS, O	1182	1180
Ethyl hexanoate	Fruity	2.7	2.7	3.0	3.7	3.7	2.0	2.9	LRI, MS, O	1236	1228
Acetoin	Butter, cream	4.0	2.3	3.7	4.0	3.7	3.3	3.5	LRI, MS, O	1304	1302
Trans-2-undecienol	Floral, rose, fruity	0.0	0.7	0.0	1.3	0.7	1.0	0.6	LRI, MS, O	1895	1899
2-Tridecanol	Leather, spices	0.0	0.7	0.0	0.0	1.0	0.0	0.3	LRI, MS, O	2312	2310
Ethyl lactate	Fruit, cream	1.0	0.0	0.0	1.3	1.0	1.0	0.7	LRI, MS, O	1334	1335
N-Hexanol	Herbaceous plant	3.7	0.0	2.3	1.3	1.0	2.0	1.7	LRI, MS, O	1371	1351
Ethyl caprylate	Fruity, creamy	3.3	1.0	0.0	1.0	0.0	0.7	1.0	LRI, MS, O	1424	1429
Acetic acid	Vinegar	4.0	4.0	4.0	4.0	3.7	4.0	3.9	LRI, MS, O	1451	1445
N-octanol	Citrus	3.7	3.7	2.0	4.0	2.7	3.7	3.3	LRI, MS, O	1559	1559
2,3-Butanediol	Creamy, fruity	2.0	1.0	1.7	2.0	0.0	0.0	1.1	LRI, MS, O	1544	1568
3-Methyl-2-butanol	Apple	1.0	0.0	0.0	1.7	0.7	0.0	0.6	LRI, MS, O	1089	1078
4-Hydroxybutyrate lactone	Floral, pollen	2.0	1.4	2.1	2.6	3.0	2.6	2.3	LRI, MS, O	1665	1665
Diethyl succinate	Floral, apple	1.7	0.7	4.0	3.7	4.0	3.3	2.9	LRI, MS, O	1686	1666
3- Methyl thiopropanol	Baked potatoes, onions	3.7	3.7	4.0	4.0	4.0	4.0	3.9	LRI, MS, O	1721	1699
Hexanoic acid	Sweat, cheese	3.3	2.3	3.7	3.7	3.7	2.7	3.2	LRI, MS, O	1842	1836
Benzyl alcohol	Flowers	0.0	0.0	0.7	0.7	1.0	1.0	0.6	LRI, MS, O	1869	1857
Phenethyl alcohol	Roses, floral	4.0	4.0	4.0	4.0	4.0	4.0	4.0	LRI, MS, O	1898	1889
2-Oxocycloheptanecarboxylic acid	Spices	2.0	2.0	3.7	1.0	2.3	2.0	2.2	LRI, MS, O	——	1826
Diethyl malate	Caramel, jam	4.0	4.0	4.0	3.3	4.0	3.7	3.8	LRI, MS, O	2035	2030
Octanoic acid	Fat, cream	4.0	3.3	2.7	4.0	4.0	3.7	3.6	LRI, MS, O	2092	2050
Isopropyl palmitate	Caramel	1.3	1.0	0.0	3.3	0.0	1.0	1.1	LRI, MS, O	——	2023
1-Methyl-4-hydroxystearate	Bitterness	0.0	0.0	1.0	2.3	2.3	1.3	1.2	LRI, MS, O	——	2239
Docosalidene enanthate	Bitterness	1.7	0.0	0.0	1.3	0.0	3.0	1.0	LRI, MS, O	——	1604
Geranyl isovalerate	Herbal incense, herbs	4.0	4.0	4.0	4.0	4.0	4.0	4.0	LRI, MS, O	1925	1923
Carboxybutylide	Caramel	1.3	2.7	1.3	0.0	1.3	0.0	1.1	LRI, MS, O	——	1950
β- Violet alcohol	Sweet	1.0	0.0	1.3	0.0	1.3	1.0	0.8	LRI, MS, O	——	1975
4-Hydroxyphenethyl alcohol	Fruity, sweet	2.3	0.0	0.0	0.0	1.3	0.0	0.6	LRI, MS, O	——	1998
2,4-Di-tert-butylphenol	Phenol	1.0	3.3	1.3	3.7	1.3	2.0	2.1	LRI, MS, O	2321	2139
Monoethyl succinate	Smoky	0.7	2.3	0.7	1.3	2.3	1.0	1.4	LRI, MS, O	——	2350
(13Z)-13-Eicosaenoic acid	Roast	1.7	0.0	0.0	0.0	0.0	0.0	0.3	LRI, MS, O	——	2365
Ethyl linoleate	Fat	1.0	0.0	0.0	0.0	0.0	0.0	0.2	LRI, MS, O	2515	2520
(5E)-5-Octadexacarbonyl	Burnt paste	0.0	0.0	0.0	0.7	0.0	0.0	0.1	LRI, MS, O	——	2593
Erucic acid	Scorched	0.7	0.0	1.0	0.0	1.0	1.0	0.6	LRI, MS, O	——	2546
Octadecanedioic acid	Smoky	0.0	0.0	0.7	1.0	0.7	1.0	0.6	LRI, MS, O	——	2523
Ethyl vanillarate	Smoky	0.0	0.0	1.0	0.0	1.0	0.0	0.3	LRI, MS, O	2676	2674
Ethyl hydroxycinnamate	Smoky	0.7	1.0	0.0	1.0	1.0	2.7	1.1	LRI, MS, O	——	2658
1,3-Glyceryl distearate	Burnt, baked	0.0	1.0	1.0	2.3	1.0	2.3	1.3	LRI, MS, O	——	2643
2-Hydroxyarmyric acid	Smoky	1.7	1.0	1.0	2.3	1.0	1.0	1.3	LRI, MS, O	——	2764
Palmitic acid	Fat	0.0	0.0	0.3	0.0	0.0	0.0	0.0	LRI, MS, O	2876	2878
Unknown compound 1	Mushroom	4	3.3	3.7	3.2	2.4	2.2	3.1	O	——	——
Unknown compound 2	Coal tar	3.2	2.3	2.7	2.1	3.8	3.4	2.9	O	——	——
Unknown compound 3	Baked potatoes	3.3	2.4	2.2	1.3	1.8	1.0	2.0	O	——	——
Unknown compound 4	Jujube, floral	3.1	2.3	2.0	3.2	2.1	3.2	2.7	O	——	——
Unknown compound 5	Sweet	2.9	3.1	2.1	3.2	3.0	1.0	2.6	O	——	——
Unknown compound 6	Caramel	3.5	2.3	3.5	3.3	2.1	3.0	3.0	O	——	——
Unknown compound 7	Green peppers, eucalyptus leaves	3.3	2.5	3.0	3.6	3.2	3.4	3.2	O	——	——
Unknown compound 8	Sweet	3.6	3.3	3.4	3.7	3.0	4.0	3.5	O	——	——

LRI: linear retention indices on the DB-Wax column obtained from the NIST Chemistry WebBook (https://webbook.nist.gov/ (accessed on 23 July 2023)). LRI *: linear retention indices calculated according to the retention times of C8-C20 n-alkanes and the retention time of each compound on the DB-Wax column [32].

**Table 3:** Aroma substances with OAV > 1 in Merlot wines.

Number	VOCs	Threshold/(μg·L^−1^) [33,34,35,36]	Average Concentration/(μg·L^−1^)	OAV
1	Ethyl butyrate	549	663.72	1.21
2	Isoamyl acetate	30	6149.58	204.99
3	Ethyl hexanoate	593	20,928.10	35.29
4	Methyl octanoate	200	724.21	3.62
5	Ethyl caprylate	874	202,743.98	231.97
6	Ethyl caprate	200	129,081.35	645.41
7	Ethyl laurate	1500	13,988.99	9.33
8	Phenyl ethyl acetate	73	532.89	7.30
9	Isoamyl alcohol	30,000	52,191.95	1.74
10	Phenethyl alcohol	10,000	62,285.16	6.23
11	β-Damascenone	0.05	120.81	2416.22
12	Hexanoic acid	420	581.67	1.38
13	Octanoic acid	500	4490.18	8.98
14	Styrene	730	795.33	1.09

**Table 4:** VOCs used in aroma reconstruction.

Serial Number	Aromatic Substances	Content/(mg·L^−1^)
1	Ethyl butyrate	1.32
2	Isoamyl acetate	4.47
3	Ethyl hexanoate	35.50
4	Methyl octanoate	1.23
5	Ethyl caprylate	412.51
6	Ethyl caprate	331.36
7	Ethyl laurate	42.85
8	Phenyl ethyl acetate	0.30
9	Isopentyl alcohol	41.44
10	Phenethyl alcohol	39.25
11	β-Damascenone	0.21
12	Hexanoic acid	1.27
13	Octanoic acid	14.38
14	Styrene	1.76
15	Acetoin	2.33
16	N-octanol	1.63
17	3-Methyl thiopropanol	0.97
18	Diethyl malate	0.60
19	Geranyl isovalerate	0.56

**Table 5:** Omission tests of Merlot wine.

Aroma Characteristic	Compound	Correct Number in All	Significance
herbal odors	geraniol isovalerate	14/20	*

The asterisk indicates significance at *p* ≤ 0.05.

**Table 6:** Basic information of three-region wines.

Sample Name	Region	Brand	Bottle	Vintage
HL1	EFHM	Imperial Horse	6	2020
HL2	EFHM	Silver Heights	6	2020
HL3	EFHM	Hongfeng Winery	6	2020
HL4	EFHM	Xinhuibin winery	6	2020
HL5	EFHM	Yuanshi Vineyard	6	2020
HL6	EFHM	Chateau Yunmo Greatwall	6	2020
XJ1	XJ	Silk Road Vineyards	6	2020
XJ2	XJ	Manasi winery	6	2020
XJ3	XJ	Tiansai Winery	6	2020
GS1	GS	Guofeng Winery	6	2020
GS2	GS	Mogao Winery	6	2020
GS3	GS	Zixuan Winery	6	2020

**Table 7:** Oenological parameters of three-region wines.

Sample Name	Region	Alcohol (%, *v*/*v*)	Dry Matter (g·L^−1^)	Titratable Acid (g·L^−1^)	Residual Sugar (g·L^−1^)	Volatile Acidity (g·L^−1^)	pH
HL1	EFHM	15.15 ± 0.18 bc	33.50 ± 0.57 c	7.16 ± 0.26 a	3.83 ± 0.01 a	0.50 ± 0.01 a	3.63 ± 0.00 j
HL2	EFHM	15.27 ± 0.02 b	29.95 ± 0.78 e	4.59 ± 0.25 de	3.25 ± 0.01 b	0.29 ± 0.01 e	3.78 ± 0.01 ef
HL3	EFHM	14.45 ± 0.01 d	28.95 ± 0.35 e	4.59 ± 0.25 de	3.13 ± 0.04 bc	0.33 ± 0.02 d	3.77 ± 0.00 f
HL4	EFHM	15.59 ± 0.00 a	33.75 ± 2.76 c	4.77 ± 0.00 de	3.74 ± 0.06 a	0.22 ± 0.01 f	3.74 ± 0.01 g
HL5	EFHM	14.62 ± 0.19 d	30.45 ± 0.35 de	6.43 ± 0.26 b	3.75 ± 0.07 a	0.34 ± 0.01 d	3.70 ± 0.00 i
HL6	EFHM	14.94 ± 0.19 c	39.20 ± 0.57 a	6.61 ± 0.00 b	3.00 ± 0.07 c	0.21 ± 0.01 f	3.72 ± 0.01 h
XJ1	XJ	14.60 ± 0.04 d	33.10 ± 0.00 c	5.33 ± 0.26 c	3.38 ± 0.06 b	0.34 ± 0.01 d	3.83 ± 0.00 c
XJ2	XJ	12.83 ± 0.00 g	36.50 ± 0.00 b	4.59 ± 0.25 de	1.63 ± 0.04 e	0.42 ± 0.03 b	3.81 ± 0.01 d
XJ3	XJ	13.33 ± 0.01 f	29.20 ± 0.00 e	4.59 ± 0.25 de	3.30 ± 0.00 b	0.45 ± 0.01 b	3.78 ± 0.00 e
GS1	GS	13.66 ± 0.17 e	32.15 ± 0.21 cd	4.41 ± 0.00 b	2.13 ± 0.18 d	0.22 ± 0.02 f	3.89 ± 0.00 b
GS2	GS	12.28 ± 0.01 h	26.30 ± 0.00 f	4.22 ± 0.26 e	1.63 ± 0.18 e	0.35 ± 0.01 cd	3.80 ± 0.00 d
GS3	GS	15.24 ± 0.00 b	32.30 ± 0.00 cd	5.51 ± 0.00 c	3.05 ± 0.07 c	0.37 ± 0.03 cd	3.91 ± 0.00 a

The data are presented in the form of “mean ± standard deviation”. In the columns, different letters represent significant differences between samples (Duncan’s test, *p* < 0.05).

## Data Availability

Not applicable.

## Supplementary Materials

The following supporting information can be downloaded at: https://www.mdpi.com/article/10.3390/molecules28155682/s1, Table S1: Calibration curves of the standards determined by HS-SPME-GC-MS.

## Sample Availability

Samples of the compound geranyl isovalerate in different concentrations are available from the authors.
