# Supplementary material for: Typical Aroma of Merlot Dry Red Wine from Eastern Foothill of Helan Mountain in Ningxia, China
Source: Molecules. 2023 Jul 27;28(15):5682. doi: 10.3390/molecules28155682 (PMC10420285; doi:10.3390/molecules28155682)
Supplement: Supplementary file 1 [file molecules-28-05682-s001.zip › molecules-2483705-supplementary.pdf]

Table S1 Calibration curves of the standards determined by HS-SPME-GC-MS

Groups	VOCs	CAS	Calibration curve	R^2
Acetate esters	Ethyl acetate	141-78-6	y = 48885.00x - 2075.60	0.9969
	Isoamyl acetate	123-92-2	y = 6969.60x - 62.99	0.9991
	Hexyl acetate	142-92-7	y = 62.87x + 0.86	0.9922
	Phenyl ethyl acetate	103-45-7	y = 74.26x + 133.84	0.9931
Ethyl esters	Ethyl butyrate	105-54-4	y = 286.02x + 1.01	0.9996
	Ethyl hexanoate	123-66-0	y = 269.63x - 183.83	0.9789
	Ethyl caprylate	106-32-1	y = 189.09x+68.07	0.9916
	Ethyl pelanoate	123-29-5	y = 110.70x + 17.12	0.9962
	Ethyl caprate	110-38-3	y = 225.10x + 776.30	0.9928
	Diethyl succinate	123-25-1	y = 843.29x + 335.01	0.9921
	Ethyl -9-decenoate	67233-91-4	y = 248.40x+368.01	0.9995
	Ethyl laurate	106-33-2	y = 219.33x + 81.21	0.9569
	Ethyl palmitate	628-97-7	y = 250.09x + 22.76	0.9651
	Ethyl lactate	97-64-3	y = 16202x - 13.28	0.9974
Other esters	Methyl octanoate	111-11-5	y = 28.99x + 1.68	0.9951
	Methyl salicylate	119-36-8	y = 183.28x + 6.2524	0.9912
Straight-chain fatty alcohols	N-Decyl alcohol	112-30-1	y = 604.28x-0.04	0.9992
Branched-chain fatty alcohols	Isopentyl alcohol	123-51-3	y = 234734.00x – 15510.00	0.9856
	2,3-Butanediol	513-85-9	y = 148,492.00x-365.28	0.9992
C6 alcohols	1-Hexanol	111-27-3	y = 1658.10x - 202.20	0.9991
Aromatic alcohols	Benzyl alcohol	100-51-6	y = 6,486.40x+1,864.40	0.9988
	Phenethyl alcohol	60-12-8	y = 3431.3x + 9273.6	0.9940
Ketones	β-Damascenone	23726-93-4	y = 7.17x + 3.85	0.9944
Fatty aldehydes	Nonanal	124-19-6	y = 187.55x + 0.44	0.9967
	Decanal	112-31-2	y = 199.90x + 2.78	0.9890
Aromatic aldehydes	Benzaldehyde	100-52-7	y = 74.83x + 5.78	0.9949
	Phenylacetaldehyde	122-78-1	y = 202.60x+12.32	0.9949
Fatty acids	Hexanoic acid	142-62-1	y = 1197.40x + 511.44	0.9901
	Octanoic acid	124-07-2	y = 481.69x + 307.82	0.9887
Aromatic alkenes	Styrene	100-42-5	y = 126.48x+4.84	0.9996
